# Response of Nutritional Values and Gut Microbiomes to Dietary Intake of ω-3 Polyunsaturated Fatty Acids in *Tenebrio molitor* Larvae

**DOI:** 10.3390/insects16090970

**Published:** 2025-09-16

**Authors:** Aifen Yang, Yiting Ye, Qiwei Liu, Jingjing Xu, Ruixi Li, Mingfeng Xu, Xiu Wang, Sida Fu, Rongrong Yu

**Affiliations:** 1 College of Life and Environmental Sciences, Hangzhou Normal University, Hangzhou 311121, China; aifen_yang@hznu.edu.cn (A.Y.); 2024210301016@stu.hznu.edu.cn (Y.Y.); 2023112010020@stu.hznu.edu.cn (Q.L.); 2022210301202@stu.hznu.edu.cn (J.X.); 2023210301158@stu.hznu.edu.cn (R.L.); zjxmf@163.com (M.X.); 2 School of Advanced Materials Engineering, Jiaxing Nanhu University, Jiaxing 314001, China; 3 College of Biological and Chemical Engineering, Jiaxing University, Jiaxing 314001, China; 4 The First Affiliated Hospital of Wenzhou Medical University, Wenzhou 325000, China

**Keywords:** *Tenebrio molitor* larvae, mealworms, gut microbial analysis, ω-3 polyunsaturated fatty acids, nutritional values

## Abstract

Due to their high nutritional value and a lower environmental impact, *Tenebrio molitor* (*T. molitor*) larvae are regarded as an alternative protein and lipid source in food industries, animal husbandry, and fishery. This study aimed to investigate the effect of ω-3 PUFA intake on the nutritional value and gut microbiota of *T. molitor* larvae. *Tenebrio molitor* (*T. molitor*) larvae were reared with wheat bran at 20–32 °C for 4 weeks to screen for a suitable temperature. EPA ethyl esters (EE), DHA ethyl esters (ED), DHA triglycerides (TG), and krill oil (KO) were supplemented in wheat bran to rear larvae for 4 weeks, and the compositions including moisture, carbohydrates, crude protein, and crude fats were analyzed. Gut microbiome was analyzed using 16S rRNA amplicon sequencing. Larvae reared on wheat bran showed optimal growth at 28 °C. ω-3 PUFA supplements increased crude protein (1.07–1.16 fold) and crude fat (1.12–1.22 fold) contents without affecting survival. Gut microbiota composition shifted significantly in all ω-3 supplemented groups, altering over 10 genera. Bacteria with changed abundance (e.g., Clostridium), known for roles in protein/lipid metabolism, likely contributed to the enhanced nutritional contents. These findings demonstrate the benefits of ω-3 PUFA supplementation in *T. molitor* rearing and identify associated gut bacteria.

## Simple Summary

*Tenebrio molitor* (*T. molitor*) larvae, commonly consumed as edible insects, are an excellent source of protein and lipids. ω-3 polyunsaturated fatty acids (PUFAs) exhibit anti-inflammatory and antioxidant properties and provide various health benefits. In this work, ω-3 PUFAs, including EPA ethyl esters (EE), DHA ethyl esters (ED), DHA triglycerides (TG), and krill oil (KO), were added as feed supplements rearing *T. molitor* larvae. Protein and fat contents were improved by ω-3 PUFA supplements. Moreover, ω-3 PUFA supplements significantly changed gut microbial community of *T. molitor* larvae. The changed gut bacteria might be involved in protein/lipid metabolism. This work highlights the potential of ω-3 PUFAs as feed supplements for *T. molitor* larvae.

## 1. Introduction

The consumption of edible insects by humans dates back to prehistoric times [1]. More than 2000 species of insects have been identified as edible insects in the world [2]. *Tenebrio molitor* (*T. molitor*) is one of the most promising and studied edible insects. The immature forms of *T. molitor* (larvae), also known as mealworms and yellow mealworms, are preferred due to their rich nutritional value. Larvae of *T. molitor* can be consumed whole or in frozen, dried, or powder forms. *Tenebrio molitor* (*T. molitor*) larvae have been authorized as human food by the European Food Safety Authority (EFSA) [3,4,5] and the Korean Food and Drug Administration (KFDA) [6]. More and more processed food products supplemented with *T. molitor* larvae, such as breads [7], biscuits [8,9], and snacks [10], are being developed.

*Tenebrio molitor* larvae serve as an alternative source of protein to traditional protein sources such as eggs, milk, and beef. Insect farming consumes less water and energy than livestock farming, indicating its excellent feed conversion ratio [11]. *Tenebrio molitor* larvae are abundant in unsaturated fatty acids, and oils derived from *T. molitor* larvae have the potential to be used in the medical, cosmetic, and food industries [12]. Soybean meal has become the dominant protein source in animal feeding. As world populations grow, protein intake and demand increase, resulting in soybean meal price fluctuations. *Tenebrio molitor* larvae have been reported as a sustainable alternative protein source to soybean meal in animal farming [13]. A recent report suggests that *T. molitor* powder shows great potential in the enhancement of gelation properties of myofibrillar protein derived from *Penaeus vannamei* [14].

Omega-3 polyunsaturated fatty acids (ω-3 PUFAs) include α-linolenic acid (ALA; 18:3), eicosapentaenoic acid (EPA; 20:5), and docosahexaenoic acid (DHA; 22:6), which cannot be synthesized by the human body and must be obtained from diets. ω-3 PUFAs exert various health benefits including anti-inflammatory [15] and antioxidant [16] properties, and they are essential for brain health [17] and effective in protecting against cancers [18], cardiovascular diseases [19], neurological disorders [20], and metabolic disorders [21]. EPA and DHA are the most intensively studied ω-3 PUFAs, and their consumption occurs mainly through the intake of fish oils and krill oils (KOs). Fish oil products contain EPA and DHA that are mainly bound to triglycerides or ethyl esters. In KO products, DHA and EPA are esterified with phospholipids (PL). The bioavailability of DHA and EPA has been extensively studied, which primarily depends on their forms. DHA and EPA in PL forms have a higher bioavailability than those in TG and EE forms [22].

*Tenebrio molitor* larvae can synthesize ALA; however, EPA and DHA are not detected in *T. molitor* larvae [23,24]. Emerging evidence has suggested that the composition of fatty acids in *T. molitor* larvae can be influenced by diet, and EPA and DHA can be detected in *T. molitor* larvae after rearing them with EPA/DHA-enriched diets [24,25]. Moreover, the EPA/DHA-enriched fish discards improve the mortality and nutritional value of *T. molitor* larvae [25]. The present study is focused on whether rearing with DHA or EPA is beneficial for the survival and nutritional values of *T. molitor* larvae and whether the diets can influence the microbial community structure of *T. molitor* larvae.

## 2. Materials and Methods

### 2.1. Mealworm Rearing and Experimental Design

*Tenebrio molitor* larvae were purchased from the Shandong Yellow Mealworm Breeding Base (Shandong, China). Wheat bran was also purchased from the Shandong Yellow Mealworm Breeding Base and used as the control diet. *Tenebrio molitor* larvae (N = 50/container) were reared in plastic trays (5.5 × 5.5 × 4.5 cm) and maintained at a relative humidity of 60% ± 5% in a dark environment.

#### 2.1.1. Experiment 1—Effect of Temperature

To screen for a suitable temperature for the growth of larvae, the larvae (approximately 4–5 weeks old) were randomly divided into four groups and they were reared at different temperatures (20, 24, 28, or 32 °C) and fed the control diet. Twelve containers were divided into four groups, with each group having three replicates to ensure accuracy and reliability in the results. The tests were conducted over a duration of 4 weeks. The number of alive larvae and their weight were recorded weekly. The survival rate and biomass increment of larvae were calculated according to the following equations:

Survival rate = the number of alive larvae/the number of larvae initially added × 100%(1)

Biomass increment = (total weight of larvae after feeding − total weight of larvae initially added)/total weight of larvae initially added × 100%(2)

At the weekly measurement, residual bran in containers was replaced by new bran. The amount of new bran was equal to that given in the first week.

#### 2.1.2. Experiment 2—Effect of EPA/DHA-Enriched Diet

To eliminate residual wheat bran in larval digestive systems, the larvae (approximately 4–5 weeks old) underwent a 48 h fasting period before the feeding experiments were conducted. Five groups were included in the subsequent experiment, including the group fed the control diet (Con) and four groups fed wheat bran supplemented with fish oil enriched with EPA ethyl esters (EE), DHA ethyl esters (ED), or DHA triglycerides (TG), or KO enriched with phospholipid DHA and EPA at the concentration of 10% (*w*/*w*). Fifteen containers were divided into five groups, with each group having three replicates to ensure accuracy and reliability in the results. Residual diets in containers were replaced by new diets weekly. The amount of new diet was equal to that given in the first week. After 4 weeks of feeding, the number of live larvae was recorded, and the survival rate was calculated. The frass from one breeding container was collected as one replicate for the gut microbiome analysis (three replicates/group), and larvae were collected for the determination of moisture, carbohydrate, crude protein, and crude fat contents. The larvae samples were stored at −20 °C for further use. The breeding containers of larvae possessed a sieve and a tray for the separation of frass. After slight shaking, the frass samples were separated and then stored at −80 °C. EE (90%), ED (90%), TG (90%), and KO were obtained from the Skuny Bioscience Co., Ltd. (Chengdu, China). Table 1 shows the protein and fat content of all diets.

### 2.2. Determination of Moisture Content

*Tenebrio molitor* larvae were cryogenically homogenized in liquid nitrogen. Pre-cleaned glass containers were dried in an oven at 105 °C for 1 h and then cooled in a desiccator for 0.5 h. The procedure was repeated until the containers were dried to a constant mass. Homogenized powder samples (1.00 g, approximately 20 larvae) were also subjected to the above cycles of drying-cooling procedures to achieve a constant mass. The moisture content of larvae was calculated using the formula: Moisture content (g/100 g fresh larvae) = (The weight of the dried glass container with undried samples − The weight of the dried glass container with dried samples)/(The weight of the dried glass container with undried samples − The weight of the dried glass container without samples) × 100. The analyses were conducted in triplicate.

### 2.3. Determination of Carbohydrate Content

Samples (0.25 g, approximately 5 larvae) were hydrolyzed by concentrated hydrochloric acid for 3 h to convert carbohydrates into reducing sugars. Total carbohydrates were quantified by the phenol-sulfuric acid method. The analyses were conducted in triplicate.

### 2.4. Determination of Crude Protein Content

Samples (2.00 g, approximately 40 larvae) were digested with concentrated sulfuric acid and a catalyst mixture containing potassium sulfate and copper sulfate at 420 °C, converting organic nitrogen into ammonium sulfate. The digestive samples were diluted with distilled water. An automatic Kjeldahl Apparatus was used to conduct nitrogen determination, and then the crude protein content of the samples was calculated. The analyses were conducted in triplicate.

### 2.5. Determination of Crude Fat Content

Samples (2.00 g, approximately 40 larvae) were mechanically disrupted, followed by acid hydrolysis with hydrochloric acid to release fat. After cooling, the crude fat was extracted with petroleum ether using the Soxhlet continuous reflux apparatus. The residues were weighed after the solvent evaporated. The analyses were conducted in triplicate.

### 2.6. Gut Microbiome Analysis

Total genomic DNA was extracted from the frass samples using the MagPure Soil DNA LQ Kit (Magen, Guangzhou, China) as per the manufacturer’s protocols. DNA concentration was measured by the NanoDrop 2000 spectrophotometer (ThermoFisher, Waltham, MA, USA). Amplification of the V3-V4 regions of the 16S rRNA gene was performed with the 343F (5′-TACGGRAGGCAGCAG-3′) and 798R (5′-AGGGTATCTAATCCT-3′) primers targeting the hypervariable regions. Agencourt AMPure XP beads were used to purify PCR products. The concentration of PCR products was quantified using the Qubit dsDNA assay kit (ThermoFisher, USA) and adjusted for sequencing. The Illumina NovaSeq6000 platform (Illumina Inc., San Diego, CA, USA) was employed to perform sequencing. The adapter was trimmed using the Cutadapt software (v1.9.3). The resulting paired-end sequences were processed using DADA2 with the default parameters of QIIME2 to remove low-quality sequences, denoise, join, and cut off chimeric sequences, and then the representative reads and the abundance table of amplicon sequence variants (ASVs) were output. The representative reads of each ASV were selected using the QIIME 2 package and aligned based on the Silva database (Version 138). Alpha and beta diversity analyses were performed by QIIME software (v 1.9.0). The linear discriminant analysis (LDA) effect size (LEfSe) method was used to compare the taxonomy abundance spectrum.

### 2.7. Statistical Analysis

Normality and homogeneity of variation were checked using the Shapiro–Wilk’s test and the Brown-Forsythe test, respectively. All data met the normality and equal variance assumptions and were analyzed using the one-way or two-way analysis of variance (ANOVA) followed by Tukey’s post hoc test. Statistical analysis was performed using the Graphpad Prism software (Version 9.0.0, Graphpad Software, LLC, Boston, MA, USA). Data are presented as the mean ± standard deviation (SD). *p* values < 0.05 were considered statistically significant.

## 3. Results

### 3.1. Effect of Temperature on the Growth of T. molitor Larvae

**Figure 1:**
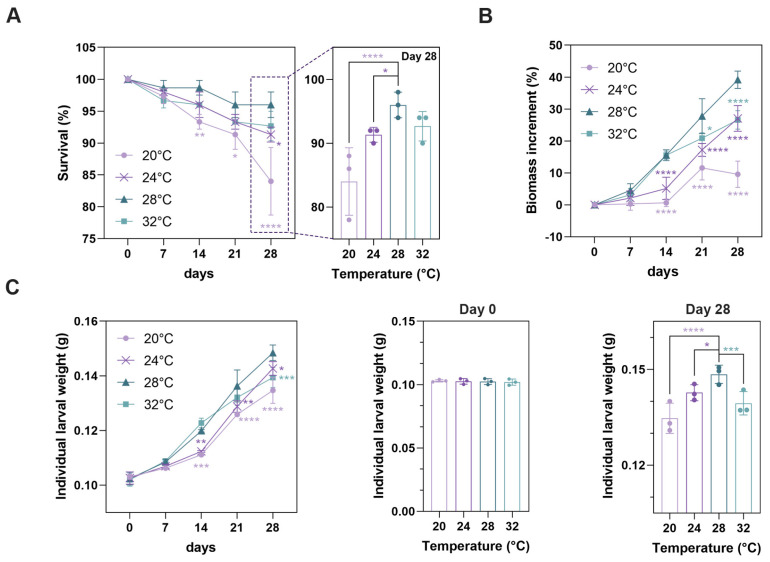
Effect of temperature on the growth performance of *T. molitor* larvae reared with the control diet. The survival rate (**A**), biomass increment (**B**), and individual weight (**C**) of larvae were analyzed. * *p* < 0.05, ** *p* < 0.01, *** *p* < 0.001, and **** *p* < 0.0001 compared with the survival rate, biomass increment, and individual weight of larvae reared at 28 °C. Data are presented as mean ± SD.

To screen a suitable temperature for the growth of *T. molitor* larvae, we incubated *T. molitor* larvae at different temperatures (20, 24, 28, and 32 °C). The survival rate of larvae was continually reduced from day 0 to day 28 when they were incubated at 20 °C (Figure 1A and Figure S1A). The survival rate of larvae remained stable from day 21 to day 28 at 28 °C (Figure 1A and Figure S1A). A slight reduction was shown in the survival rate of larvae at 24 °C and 32 °C from day 21 to day 28 (Figure 1A and Figure S1A). During the 28-day incubation period, the highest survival rate of larvae was observed at 28 °C (28 °C: 96.00% ± 2.00%; 20 °C: 84.00% ± 5.29%; 24 °C: 91.33% ± 1.15%; 32 °C: 92.67% ± 2.31%; Figure 1A). Biomass of larvae was increased from day 14 at 28 and 32 °C, while biomass of larvae was not significantly increased until day 21 at 20 and 24 °C (Figure 1B). Biomass increment of larvae was significantly higher at 28 °C than that at 20, 24, and 32 °C (28 °C: 39.18% ± 2.71%; 20 °C: 9.60% ± 4.11%; 24 °C: 27.08% ± 4.02%; 32 °C: 26.60% ± 2.87%; Figure S1B). There was no significant difference in individual larval weight among all groups at day 0 (Figure 1C). After 28-day incubation, individual larval weight showed the most significant increases at 28 °C (28 °C: 0.148 ± 0.003 g; 20 °C: 0.135 ± 0.005 g; 24 °C: 0.143 ± 0.002 g; 32 °C: 0.139 ± 0.004 g; Figure 1C). Based on these findings, 28 °C was chosen for subsequent experiments.

### 3.2. Effect of Dietary Supplementation with ω-3 PUFA on the Nutritional Value of T. molitor Larvae

**Figure 2:**
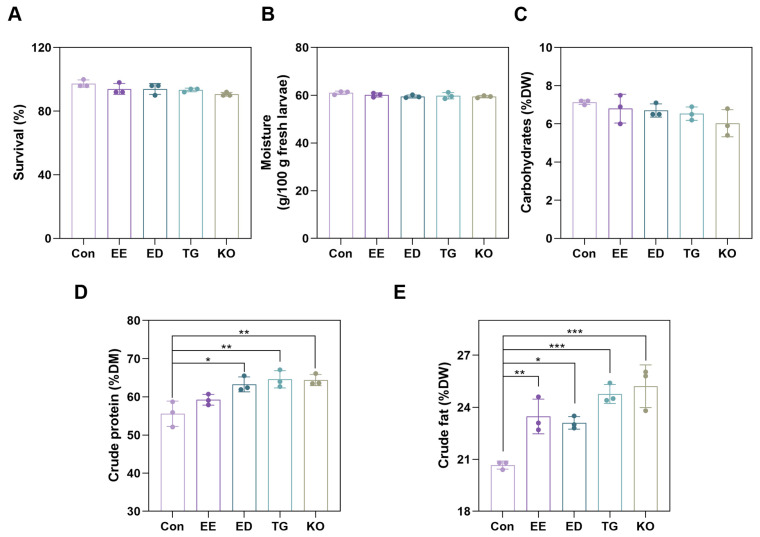
Effect of dietary inclusion of ω-3 PUFAs on the survival and the nutritional value of *T. molitor* larvae. The survival rate (**A**) of larvae was analyzed, and the moisture (**B**), carbohydrate (**C**), crude protein (**D**), and crude fat (**E**) content of larvae were measured. * *p* < 0.05, ** *p* < 0.01, and *** *p* < 0.001. Data are presented as mean ± SD. Con, control; EE, EPA ethyl esters; ED, DHA ethyl esters; TG, DHA triglycerides; KO, krill oil; DM, dry matter.

Dietary ω-3 PUFAs have been proven to offer benefits for health. *Tenebrio molitor* larvae were reared with different diets supplemented with enriched with EE, ED, or TG or KO. The survival rate of larvae was not significantly affected by these diets enriched with ω-3 PUFAs (Figure 2A). The nutritional value of larvae was also evaluated. Diets supplemented with ω-3 PUFAs had no significant impact on the moisture and carbohydrate content of larvae (Figure 2B,C). The crude protein and the crude fat content of larvae varied among different groups. As shown in Figure 2D, the crude protein content of larvae in the EE group (59.23% ± 1.40% on dry weight basis) showed an increasing trend and that of larvae in the ED, TG, and KO groups (ED: 59.23% ± 1.40% on dry weight basis; TG: 63.27% ± 1.95% on dry weight basis; KO: 64.60% ± 1.47% on dry weight basis) significantly increased as compared with the control group (55.57% ± 3.31% on dry weight basis). Compared with the control group (20.67% ± 0.23% on dry weight basis), crude fat content was increased in the EE, ED, TG, and KO groups (EE: 23.47% ± 1.00% on dry weight basis, ED: 23.10% ± 0.36% on dry weight basis; TG: 24.77% ± 0.55% on dry weight basis; KO: 25.21% ± 1.23% on dry weight basis) (Figure 2E). These findings indicate that diets enriched with ω-3 PUFAs improve the nutritional value of larvae.

### 3.3. Effect of ω-3 PUFA Supplementation on the Gut Microbial Community of T. molitor Larvae

**Figure 3:**
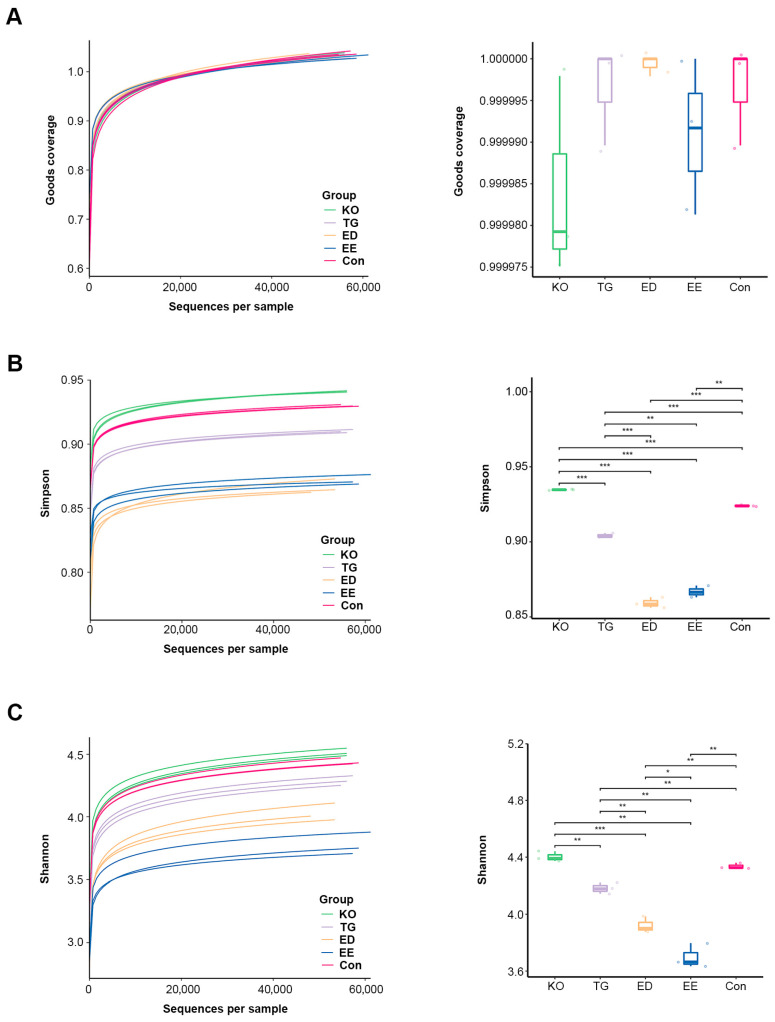
Alpha diversity analysis of the gut microbiome of *T. molitor* larvae reared with the diet supplemented with ω-3 PUFAs. Rarefaction curves and box plots of Goods coverage (**A**), Simpson diversity index (**B**), and Shannon diversity (**C**). * *p* < 0.05, ** *p* < 0.01, and *** *p* < 0.001. Con, control; EE, EPA ethyl esters; ED, DHA ethyl esters; TG, DHA triglycerides; KO, krill oil.

**Figure 4:**
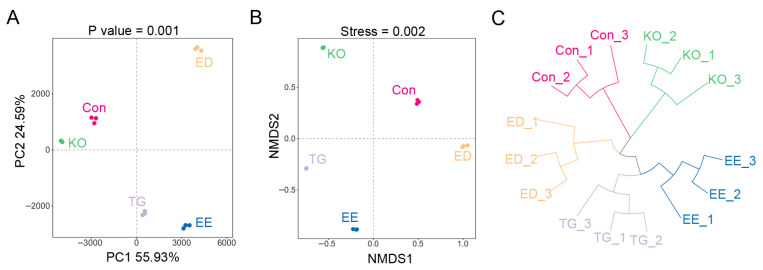
Beta diversity analysis of the gut microbiome of *T. molitor* larvae reared with the diet supplemented with ω-3 PUFAs. PCA (**A**), NMDS (**B**), and UPGMA cluster analyses (Euclidean) (**C**) were performed. PCA, Principal Coordinate Analysis. NMDS, Non-Metric Multidimensional Scaling. UPGMA, Unweighted Pair Group Method with Arithmetic Mean. Con, control; EE, EPA ethyl esters; ED, DHA ethyl esters; TG, DHA triglycerides; KO, krill oil.

The effect of different diets on microbial community structure was investigated using 16S rRNA amplicon sequencing. The alpha diversity index reflects the richness and evenness of the microbial communities for each group. As shown in Figure 3A, the goods coverage of the bacteria in all samples was above 0.9999, indicating that the majority of bacteria were detected. Rarefaction curve for Simpson and Shannon index gradually reached a plateau with increasing sequencing depth (Figure 3B,C), reflecting that the sequencing was saturated. Simpson index represents the diversity of species in the sample, while Shannon index reflects the richness and evenness of species in the sample. Both the Shannon index and Simpson index in the EE, ED, and TG groups were significantly lower than those in the control and KO groups (Figure 3B,C). The results indicated that EE, ED, and TG feeding reduced species diversity in the gut of mealworms. Beta diversity was measured to analyze the difference among all the groups. Principal component analysis (PCA) revealed that the gut bacterial communities of larvae fed different diets were significantly distinct from each other (Figure 4A). Similar results were observed in NMDS analysis and UPGMA cluster analysis (Figure 4B,C).

**Figure 5:**
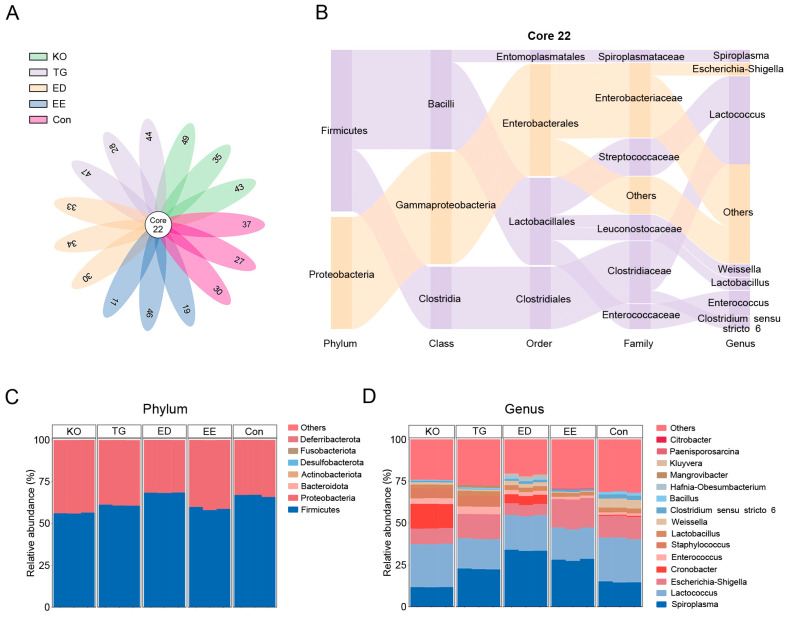
Analysis of gut microbial communities of *T. molitor* larvae reared with the diet supplemented with ω-3 PUFAs. The Venn diagram (**A**) and the Sankey diagram (**B**) show core ASVs. Stacked bar graphs display the composition of the intestinal microbiome at the phylum (**C**) and genus (**D**) levels. Con, control; EE, EPA ethyl esters; ED, DHA ethyl esters; TG, DHA triglycerides; KO, krill oil.

There were 22 core ASVs shared by the gut of the larvae fed different diets (Figure 5A). The core ASVs mainly consist of *Spiroplasma*, *Escherichia_Shigella*, *Lactococcus*, *Weissella*, *Lactobacillus*, *Enterococcus*, and *Clostridium sensu stricto 6* (Figure 5B). Changes in the composition of the intestinal microbiome at the phylum and genus levels were analyzed. *Firmicutes* and *Proteobacteria* were the core phyla that dominate the gut of the larva in the control group, accounting for 66.67% and 33.25% (Figure 5C). Increased relative abundance of *Firmicutes* and decreased relative abundance of *Proteobacteria* were shown in the ED group (Figure 5C). In contrast, the EE, TG, and KO groups showed a lower relative abundance of *Firmicutes* and a higher relative abundance of *Proteobacteria* than the control group (Figure 5C). Top15 genera made up >68% of the intestinal microbiome (Figure 5D). The composition of the gut bacterial communities of larvae varied among different groups. The predominating genus in the control group was *Lactococcus* (26.20%), followed by *Spiroplasma* (14.79%), *Escherichia_Shigella* (13.14%), *Weissella* (5.31%), *Clostridium sensu stricto 6* (2.49%), *Lactobacillus* (2.47%), *Bacillus* (1.68%), *Enterococcus* (1.45%), *Cronobacter* (0.61%), *Staphylococcus* (0.36%), and *Paenisporosarcina* (0.06%) (Figure 5D). The EE, ED, and TG groups displayed a distinct microbial profile, characterized by elevated abundance of *Spiroplasma* (EE group: 28.06%; ED group: 33.62%; and TG group: 22.58%) and decreased abundance of *Lactococcus* (EE group: 18.90%; ED group: 20.92%; and TG group: 18.04%) compared to the control group (Figure 5D). The abundance of *Lactococcus* in the KO group (25.78%) was similar to that in the control group (Figure 5D). The KO group exhibited a higher abundance of *Cronobacter* (14.60%), *Enterococcus* (3.39%), and *Staphylococcus* (6.12%) compared to the control group (Figure 5D).

**Figure 6:**
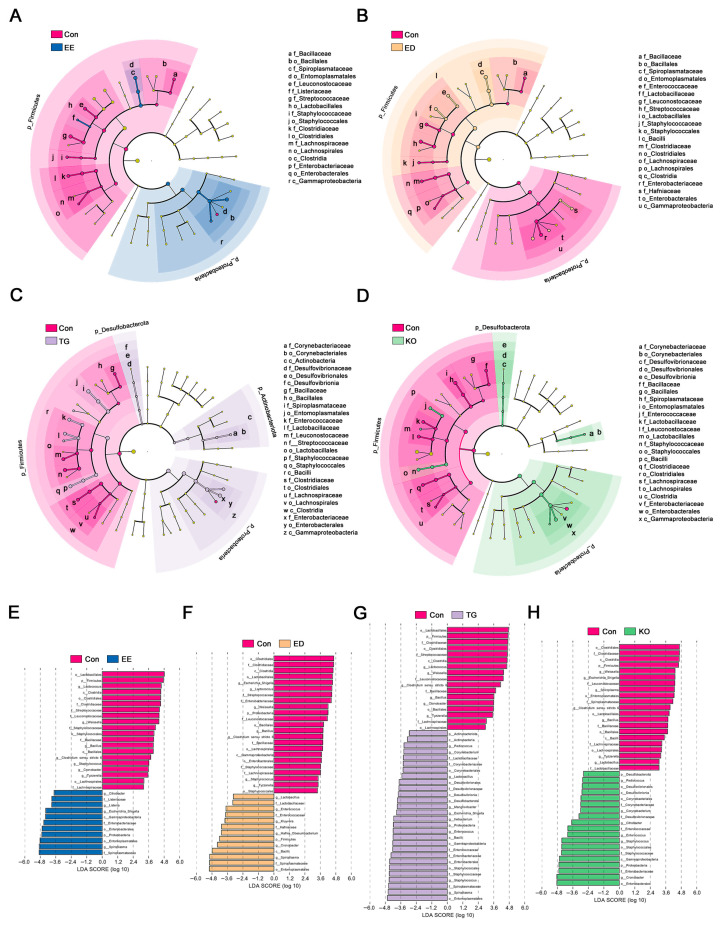
LEfSe analysis of the gut microbiome of *T. molitor* larvae reared with the diet supplemented with ω-3 PUFAs. The taxonomic cladogram and the Histogram generated from the LEfSe analysis. The taxa with an LDA score > 2 were considered biomarker taxa. LDA, Linear discriminant analysis. LEfSe, linear discriminant analysis (LDA) effect size. Con, control; EE, EPA ethyl esters; ED, DHA ethyl esters; TG, DHA triglycerides; KO, krill oil.

The LEfSe analysis was performed to evaluate the differences in the abundance of microbial communities at all taxonomic levels between the control group and the other groups. The cladogram showing taxa with LDA scores greater than 2 and *p* values less than 0.05 is presented in Figure 6A–D, and the corresponding LDA value for each lineage is shown in Figure 6E–H. LEfSe identified 11, 13, 15, and 13 altered genera in the EE, ED, TG, and KO groups, respectively. The relative abundance of *Spiroplasma*, *Escherichia_Shigella*, *Listeria*, and *Citrobacter* was higher in the EE group, while the relative abundance of *Lactococcus*, *Weissella*, *Bacillus*, *Clostridium sensu stricto 6*, *Staphylococcus*, *Cronobacter*, and *Tyzzerella* was higher in the control group (Figure 6A,E). Multiple genera were enriched in the ED group, including *Spiroplasma*, *Cronobacter*, *Hafnia_Obesumbacterium*, *Kluyvera*, *Enterococcus*, and *Lactobacillus*. Moreover, the LEfSe results demonstrated that *Escherichia_Shigella*, *Lactococcus*, *Weissella*, *Bacillus*, *Clostridium sensu stricto 6*, *Staphylococcus*, and *Tyzzerella* were significantly decreased in the ED group (Figure 6B,F). In the TG group, microbes such as *Spiroplasma*, *Staphylococcus*, *Enterococcus*, *Ileibacterium*, *Escherichia_Shigella*, *Mangrovibacter*, *Lactobacillus*, *Corynebacterium*, and *Pediococcus* had obvious advantages, while in the control group, microbes such as *Lactococcus*, *Weissella*, *Clostridium sensu stricto 6*, *Bacillus*, *Cronobacter*, and *Tyzzerella* had obvious advantages (Figure 6C,G). The indicator genera in the KO group were *Cronobacter*, *Staphylococcus*, *Enterococcus*, *Citrobacter*, *Corynebacterium*, and *Pediococcus* in the KO group, and those in the control group were *Weissella*, *Escherichia_Shigella*, *Spiroplasma*, *Clostridium sensu stricto 6*, *Bacillus*, *Tyzzerella*, and *Lactobacillus* (Figure 6D,H). These results demonstrate that diets enriched with ω-3 PUFAs induced the alteration of gut bacterial community composition.

## 4. Discussion

The optimization of the rearing temperature is an effective way to improve rearing efficiency. Rearing temperature is an important abiotic factor affecting the growth of *T. molitor* larvae. *Tenebrio molitor* larvae are commonly reared at 25–28 °C [26,27,28], while the highest and lowest rearing temperatures were 10 °C and 35 °C [29], respectively. *Tenebrio molitor* larvae derived from different geographical areas may show the best growth performance at different temperatures [30,31,32]. To obtain the best growth performance, we screened the optimal temperature for the growth of *T. molitor* larvae. We observed that the growth performance of *T. molitor* larvae was improved with the increase in temperature up to 28 °C and then that became poor at 32 °C. Low food availability by heat stress may be linked to the poor growth performance of *T. molitor* larvae at a high temperature. In fact, nutritional values of *T. molitor* larvae are also affected by temperature. *Tenebrio molitor* larvae reared at 28 °C had a higher dry matter content, ash content, and crude fat content than those reared at a lower temperature [31]. Bjørge et al. [32] found that the excessive rearing temperature had negative effects on the growth and nutritional value of *T. molitor* larvae. This work highlights the importance of optimal temperature in insect rearing.

*Tenebrio molitor* larvae are an alternative source of protein-rich animal feed [33,34,35]. In addition to being used in animal feeding, *T. molitor* larvae are a sustainable food source for humans [36]. Protein and fat extracted from *T. molitor* larvae benefit health. *Tenebrio molitor* larvae-derived protein extracts exert multiple activities, including anti-inflammatory, anti-aging, anti-obesity, and anti-hyperglycemia [37,38,39]. Fat or oil extracted from *T. molitor* larvae has been linked to anti-cancer, anti-oxidative, wound-repairing, and cholesterol-lowering effects [12,40,41]. Changes in the diet rearing *T. molitor* larvae lead to alterations in nutritional values, including protein and fat compositions [26,42]. The present work illustrated that dietary intake of ω-3 PUFAs increased the nutritional values of *T. molitor* larvae, especially crude fat contents. The fat content of diets in the TG and KO groups was similar to that of diets in the EE and ED groups; however, the increase in the crude fat content of *T. molitor* larvae was more obvious in the TG and KO groups. Bioavailability of DHA and EPA is associated with their chemical forms. TG and PL forms may also show better bioavailability than EE forms in *T. molitor* larvae, thereby resulting in more obvious increases in crude fat contents. Diets with ω-3 PUFA-enriched seed meals enhanced ω-3 PUFA contents in *T. molitor* larvae [43]. These findings highlighted that *T. molitor* larvae possessed the ability to accumulate PUFAs from dietary sources. ω-3 PUFAs provide multifaceted health benefits [44]. It merits further exploration in our future work whether dietary intake of ω-3 PUFAs has a positive effect on the biological activities of *T. molitor* larvae-derived protein/fat extracts.

Microorganisms colonize the gut of insects via foods, which play a crucial role in the digestion, metabolism, immune system function, pathogen resistance, and reproduction of hosts [45]. Changes in diets significantly affect the composition of gut microbiota in *T. molitor* larvae [46]. In this work, the gut microbial community in *T. molitor* larvae was altered by dietary intake of ω-3 PUFAs. Dietary intake of KO had little influence on species diversity, while dietary intake of EE, ED, and TG downregulated species diversity in the gut of *T. molitor* larvae. *Spiroplasma*, *Lactococcus*, *Weissella*, *Lactobacillus*, *Enterococcus*, and *Clostridium* were common bacteria in the gut of *T. molitor* larvae, which was consistent with previous studies [47,48]. Among the common bacteria, *Spiroplasma* is identified as a pathogen of insects. However, *Spiroplasma* has no harmful effect on *T. molitor* larvae [49], confirming that *Spiroplasma* in the gut does not act as a pathogen to *T. molitor* larvae. *Spiroplasma* served as the common biomarker in the gut of *T. molitor* larvae under dietary conditions with EE, ED, and TG. Gut bacteria, including *Lactococcus*, *Weissella*, *Bacillus*, and *Clostridium sensu stricto 6*, obviously decreased in *T. molitor* larvae reared with EE-, ED-, and TG-enriched diets. *Enterococcus* and *Lactobacillus* increased in *T. molitor* larvae reared with ED- and TG-enriched diets. *Cronobacter*, *Staphylococcus*, *Enterococcus*, *Citrobacter*, *Corynebacterium*, and *Pediococcus* significantly increased but *Weissella*, *Spiroplasma*, *Clostridium sensu stricto 6*, *Bacillus*, and *Lactobacillus* significantly decreased by dietary intake of KO. In addition, ED and KO diets reduced the presence of potentially pathogenic bacteria such as *Escherichia_Shigella*. Insect symbiotic microbiota produces enzymes required for food digestion and absorption in insects, thus playing an important role in the nutritional metabolism of their hosts. Many microbial species with abundance changes in the gut are involved in protein or/and lipid catabolism. *Clostridium*, *Lactobacillus*, and *Citrobacter* in the gut produce proteases involved in proteolysis [50]. Lactic acid bacteria such as *Lactobacillus*, *Lactococcus*, and *Enterococcus* influence the degradation of free amino acids [51]. The regulation of host amino acid metabolism by *Enterococcus* has been confirmed in *Clanis bilineata tsingtauica* [52]. *Citrobacter* participates in protein metabolism by interaction with intestinal proteases in *Hermetia illucens* [53]. *Bacillus* enhance protein degradation in *Hermetia illucens* larvae [54] and fat degradation in *Ectropis grisescens* [55]. *Spiroplasma* in *Glossina fuscipes* has been reported to regulate host lipid synthesis [56]. *Corynebacterium* and *Escherichia_Shigella* are of vital importance in lipid synthesis and storage in *Hermetia illucens* [57]. *Weissella* and *Pediococcus* contribute to lipid metabolism in human adipocytes or rodents [58,59]. Taken together, it is speculated that changes in the content of crude protein and fat may be linked to structural changes in gut microbiota. In addition, similar to the enhanced stability and functional performance observed in protein-polysaccharide complexes such as phycocyanin nanoparticles and cationic starch-based Pickering emulgels [60], the altered gut microbiota in *T. molitor* larvae may facilitate improved lipid and protein metabolism through analogous interfacial and structural mechanisms.

*Tenebrio molitor* can degrade and metabolize plastic wastes, graphene materials, and lignocellulose wastes through synergistic biological activities of their gut microbiota [47,61,62,63]. *Spiroplasma* has the potential to degrade polyethylene and polyethylene terephthalate in *T. molitor* [64,65]. *Cronobacter* from the gut of *T. molitor* larvae has been identified as a candidate bacterium degrading expanded polystyrene [66]. *Staphylococcus* can secrete lignin peroxidase to degrade lignocellulose in *Macrotermes nigeriense termites* [67]; and it is also closely associated with polystyrene degradation in *T. molitor* [47]. The above mentioned bacteria are affected by dietary intake of ω-3 PUFAs; however, it is unclear whether dietary intake of ω-3 PUFAs is beneficial for the biodegradation activities of *T. molitor*, which should be evaluated in the future.

## 5. Conclusions

In conclusion, the inclusion of ω-3 PUFAs in the diet showed no negative effect on the survival of *T. molitor* larvae in a 4-week trial, which also reflected the strong adaptability of *T. molitor* larvae. The dietary intake of ω-3 PUFAs exhibited the potential to increase crude protein and fat contents. Furthermore, multiple gut bacteria (e.g., *Clostridium*, *Citrobacter*, *Lactobacillus*, *Lactococcus*, *Enterococcus*, *Bacillus*, *Spiroplasma*, *Corynebacterium*, *Escherichia_Shigella*, *Weissella*, and *Pediococcus*) contributed to the gut microbiota community shift. The bacteria with abundance changes may be involved in the ingestion of protein and fat in *T. molitor* larvae. The study highlights the potential of ω-3 PUFA intake as a novel feeding strategy for *T. molitor* larvae.

## Figures and Tables

**Table 1:** The protein and fat content of all diets used for rearing *T. molitor* larvae.

Component	Con	EE	ED	TG	KO
Protein (%DM)	15.63 ± 0.43	12.43 ± 0.66 ^ns^	13.89 ± 1.80 ^ns^	12.88 ± 0.56 ^ns^	13.16 ± 1.10 ^ns^
Fat (%DM)	4.08 ± 0.29	13.33 ± 0.86 ****	13.99 ± 1.07 ****	13.34 ± 1.61 ****	13.15 ± 0.57 ****

^ns^ No significance between the EPA/DHA-enriched diet and the Con diet. **** *p* < 0.0001 compared with the fat content of Con diets. Data are presented as mean ± SD. Con, control; EE, EPA ethyl esters; ED, DHA ethyl esters; TG, DHA triglycerides; KO, krill oil; DM, dry matter.

## Data Availability

The original contributions presented in this study are included in the article/Supplementary Material. Further inquiries can be directed to the corresponding authors.

## Supplementary Materials

The following supporting information can be downloaded at: https://www.mdpi.com/article/10.3390/insects16090970/s1, Figure S1: The survival (A) and biomass increment (B) of *T. molitor* larvae reared with the control diet at different rearing temperatures in a 4-week trial. * *p* < 0.05, ** *p* < 0.01, *** *p* < 0.001, and **** *p* < 0.0001. Data are presented as mean ± SD.

## Abbreviations

The following abbreviations are used in this manuscript:

*T. molitor*	*Tenebrio molitor*
KFDA	Korean Food and Drug Administration
EFSA	European Food Safety Authority
ω-3 PUFAs	Omega-3 polyunsaturated fatty acids
ALA	α-linolenic acid
EPA	Eicosapentaenoic acid
DHA	Docosahexaenoic acid
KO	Krill oils
ASVs	Amplicon sequence variants
LDA	Linear discriminant analysis
LEfSe	Linear discriminant analysis effect size
ANOVA	Analysis of variance
PCoA	Principal co-ordinates analysis
