# Supplementary figures and images for: Response of Nutritional Values and Gut Microbiomes to Dietary Intake of ω-3 Polyunsaturated Fatty Acids in Tenebrio molitor Larvae
Source: Insects. 2025 Sep 16;16(9):970. doi: 10.3390/insects16090970 (PMC12471062; doi:10.3390/insects16090970)

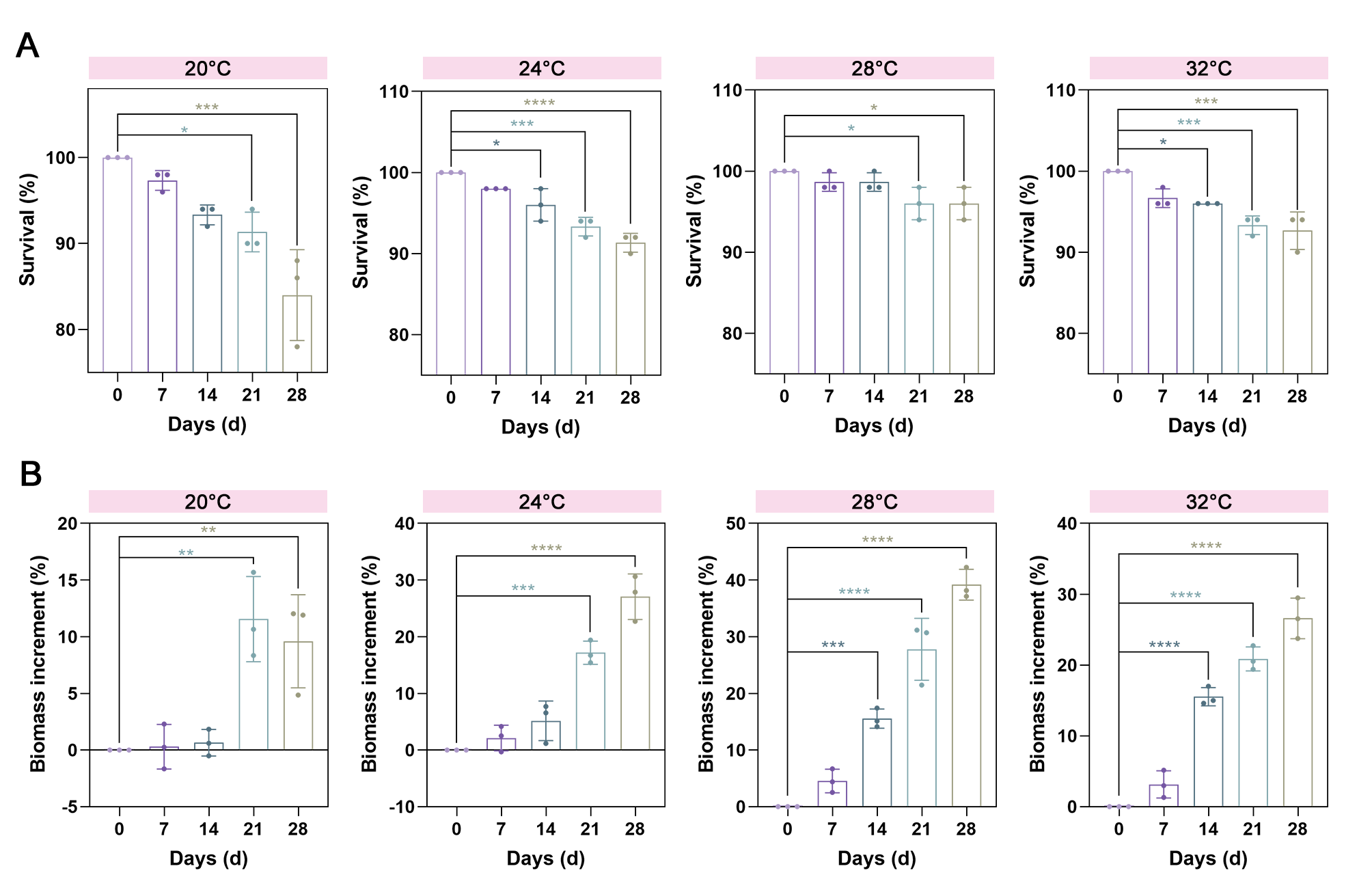

Supplement: Supplementary file 1 [file insects-16-00970-s001.zip › insects-3847316-supplementary.tif]
